# Supplementary material for: Single Cell Transcriptomic and Chromatin Profiles Suggest Layer Vb Is the Only Layer With Shared Excitatory Cell Types in the Medial and Lateral Entorhinal Cortex
Source: Front Neural Circuits. 2022 Jan 26;15:806154. doi: 10.3389/fncir.2021.806154 (PMC8826650; doi:10.3389/fncir.2021.806154)
Supplement: Supplementary file 14 [file Data_Sheet_1.ZIP › Deep_vs_Sup_Motifs/homerResults.html]

Deep\_vs\_Sup\_Motifs// - Homer de novo Motif Results


# Homer *de novo* Motif Results (Deep\_vs\_Sup\_Motifs//)

Known Motif Enrichment Results  
Gene Ontology Enrichment Results  
If Homer is having trouble matching a motif to a known motif, try copy/pasting the matrix file into
STAMP  
More information on motif finding results: HOMER
| Description of Results
| Tips
  
Total target sequences = 9297  
Total background sequences = 32569  
\* - possible false positive  

|  |  |  |  |  |  |  |  |  |
| --- | --- | --- | --- | --- | --- | --- | --- | --- |
| Rank | Motif | P-value | log P-pvalue | % of Targets | % of Background | STD(Bg STD) | Best Match/Details | Motif File |
| 1 | C G A T C A T G A T G C A G T C C G T A C T G A C T A G G C T A G C A T G C A T | 1e-201 | -4.637e+02 | 53.50% | 37.97% | 244.0bp (246.5bp) | NFIA/MA0670.1/Jaspar(0.928) More Information \| Similar Motifs Found | motif file (matrix) |
| 2 | C G A T T C A G C T G A G A T C G T C A A C G T A C G T G C A T G T C A T G C A | 1e-140 | -3.236e+02 | 32.35% | 21.07% | 250.9bp (254.0bp) | NR4A1/MA1112.2/Jaspar(0.852) More Information \| Similar Motifs Found | motif file (matrix) |
| 3 | T A G C C G A T A C T G C G T A A C G T C G T A C G T A A T C G | 1e-88 | -2.046e+02 | 40.83% | 30.98% | 257.2bp (267.6bp) | Gata1(Zf)/K562-GATA1-ChIP-Seq(GSE18829)/Homer(0.959) More Information \| Similar Motifs Found | motif file (matrix) |
| 4 | C G A T A G C T G A T C C T G A A G T C C T G A A G T C A G C T | 1e-73 | -1.695e+02 | 35.90% | 27.25% | 254.4bp (256.2bp) | TBR1/MA0802.1/Jaspar(0.844) More Information \| Similar Motifs Found | motif file (matrix) |
| 5 | T C G A C T A G T C G A G T C A T C G A C G T A G C A T A C G T A G C T A C G T | 1e-51 | -1.176e+02 | 36.50% | 29.22% | 245.6bp (263.3bp) | PB0165.1\_Sox11\_2/Jaspar(0.673) More Information \| Similar Motifs Found | motif file (matrix) |
| 6 | G A C T A G C T G A T C G T A C G T C A G T C A G T C A G C A T A G T C G A T C G A T C G C A T | 1e-49 | -1.140e+02 | 45.57% | 38.01% | 256.7bp (252.8bp) | GFY(?)/Promoter/Homer(0.612) More Information \| Similar Motifs Found | motif file (matrix) |
| 7 | C A T G C T A G T C G A C G A T A C T G C A G T C T A G C G T A C A T G T C G A G C T A T C G A | 1e-40 | -9.252e+01 | 41.43% | 34.75% | 256.1bp (259.0bp) | ZKSCAN5/MA1652.1/Jaspar(0.772) More Information \| Similar Motifs Found | motif file (matrix) |
| 8 | A G C T C G T A A C T G C T A G C G T A C G T A A C G T A G T C | 1e-39 | -9.094e+01 | 45.76% | 39.01% | 254.4bp (258.1bp) | PH0037.1\_Hdx/Jaspar(0.747) More Information \| Similar Motifs Found | motif file (matrix) |
| 9 | G C T A G C A T G T A C G C T A G T A C T G C A C T A G G T A C A G C T G T C A | 1e-38 | -8.839e+01 | 31.15% | 25.14% | 255.4bp (255.4bp) | TBX20/MA0689.1/Jaspar(0.742) More Information \| Similar Motifs Found | motif file (matrix) |
| 10 | G A T C T C A G C G A T A C T G G T A C G A T C G C T A C A T G G T A C T A C G | 1e-38 | -8.767e+01 | 31.23% | 25.25% | 249.4bp (238.4bp) | HIC1(Zf)/Treg-ZBTB29-ChIP-Seq(GSE99889)/Homer(0.858) More Information \| Similar Motifs Found | motif file (matrix) |
| 11 | A C G T T C G A C A G T A G T C T A C G T G C A G C A T G A C T G C T A G A C T | 1e-34 | -7.842e+01 | 47.20% | 40.91% | 256.3bp (266.4bp) | CUX2/MA0755.1/Jaspar(0.809) More Information \| Similar Motifs Found | motif file (matrix) |
| 12 | G T C A A G T C C G T A G T A C A G C T A G T C A C G T A C G T | 1e-30 | -6.948e+01 | 60.57% | 54.66% | 257.6bp (261.3bp) | SD0003.1\_at\_AC\_acceptor/Jaspar(0.689) More Information \| Similar Motifs Found | motif file (matrix) |
| 13 | A C G T C G T A A C G T A G T C C G A T A C G T A C G T A G T C | 1e-20 | -4.771e+01 | 8.77% | 6.26% | 264.6bp (280.5bp) | SD0003.1\_at\_AC\_acceptor/Jaspar(0.708) More Information \| Similar Motifs Found | motif file (matrix) |
| 14 | A G T C C G A T A G T C C G A T C T A G C G T A C G T A C G T A C T G A C T G A C G A T T C A G | 1e-20 | -4.675e+01 | 1.06% | 0.35% | 231.5bp (286.7bp) | SOX14/MA1562.1/Jaspar(0.632) More Information \| Similar Motifs Found | motif file (matrix) |
| 15 | A T G C G A C T C G T A A C G T T G C A T C G A A G T C A T G C | 1e-18 | -4.170e+01 | 4.98% | 3.24% | 271.9bp (244.9bp) | ZBTB12(Zf)/HEK293-ZBTB12.GFP-ChIP-Seq(GSE58341)/Homer(0.656) More Information \| Similar Motifs Found | motif file (matrix) |
| 16 | A C G T C G A T G T A C C T A G A G T C C G T A A T C G A T G C | 1e-16 | -3.800e+01 | 7.31% | 5.27% | 235.0bp (254.0bp) | Tbr1(T-box)/Cortex-Tbr1-ChIP-Seq(GSE71384)/Homer(0.673) More Information \| Similar Motifs Found | motif file (matrix) |
| 17 | A G T C A C G T A G T C C G T A A G T C C G T A A C T G C G T A A G T C A C G T A C T G A C G T | 1e-16 | -3.701e+01 | 50.61% | 46.34% | 262.0bp (251.0bp) | PR(NR)/T47D-PR-ChIP-Seq(GSE31130)/Homer(0.605) More Information \| Similar Motifs Found | motif file (matrix) |
| 18 | G A C T G T C A G C A T C T A G T C A G C T A G C T A G C G T A A G C T C A G T | 1e-15 | -3.503e+01 | 2.57% | 1.47% | 233.4bp (273.8bp) | MZF1/MA0056.2/Jaspar(0.875) More Information \| Similar Motifs Found | motif file (matrix) |
| 19 \* | C G T A C G T A G T C A A C G T A C G T A G T C A G T C C G T A C G T A A G T C C G T A A G T C | 1e-11 | -2.537e+01 | 0.14% | 0.01% | 161.4bp (0.0bp) | CEBP:AP1(bZIP)/ThioMac-CEBPb-ChIP-Seq(GSE21512)/Homer(0.673) More Information \| Similar Motifs Found | motif file (matrix) |
| 20 \* | A G T C A C T G C G T A C G T A C G T A A C T G A C G T A G T C C G T A C G T A | 1e-10 | -2.434e+01 | 0.10% | 0.00% | 183.0bp (48.9bp) | Nr2e1/MA0676.1/Jaspar(0.822) More Information \| Similar Motifs Found | motif file (matrix) |
| 21 \* | C G T A A G T C C G T A A C G T A C G T G T A C A C G T A G T C C G T A A C G T A C G T A G T C | 1e-10 | -2.363e+01 | 0.18% | 0.02% | 172.9bp (122.6bp) | PRDM1(Zf)/Hela-PRDM1-ChIP-Seq(GSE31477)/Homer(0.628) More Information \| Similar Motifs Found | motif file (matrix) |
| 22 \* | C G A T A G T C A C G T A C T G A G T C C G T A A G T C A C T G | 1e-9 | -2.210e+01 | 1.61% | 0.93% | 223.0bp (279.4bp) | HIF-1b(HLH)/T47D-HIF1b-ChIP-Seq(GSE59937)/Homer(0.729) More Information \| Similar Motifs Found | motif file (matrix) |
| 23 \* | C T A G C G A T T C A G G A C T T C A G A G C T C T A G G C A T | 1e-9 | -2.190e+01 | 55.22% | 52.01% | 277.1bp (243.7bp) | PB0130.1\_Gm397\_2/Jaspar(0.721) More Information \| Similar Motifs Found | motif file (matrix) |
| 24 \* | C T A G A T G C C G T A C T A G A G T C G T A C A T C G A G T C C G T A A T C G A G T C C G T A | 1e-8 | -2.042e+01 | 1.43% | 0.81% | 255.6bp (220.5bp) | PRDM9(Zf)/Testis-DMC1-ChIP-Seq(GSE35498)/Homer(0.730) More Information \| Similar Motifs Found | motif file (matrix) |
| 25 \* | A C T G A G T C C T A G C G T A A C T G A C T G A G T C C T A G C G T A A C T G A C T G A G T C | 1e-8 | -1.980e+01 | 39.19% | 36.26% | 267.9bp (248.3bp) | Sp1(Zf)/Promoter/Homer(0.647) More Information \| Similar Motifs Found | motif file (matrix) |
| 26 \* | A C T G G A C T G A C T G A C T T A C G C T A G A G T C A C T G | 1e-8 | -1.943e+01 | 1.13% | 0.61% | 262.0bp (278.0bp) | PB0112.1\_E2F2\_2/Jaspar(0.693) More Information \| Similar Motifs Found | motif file (matrix) |
| 27 \* | A C G T A C T G A C G T A G T C A C G T C T A G A C G T T G A C A G C T C T G A | 1e-7 | -1.822e+01 | 2.76% | 1.92% | 259.9bp (267.4bp) | Smad4(MAD)/ESC-SMAD4-ChIP-Seq(GSE29422)/Homer(0.737) More Information \| Similar Motifs Found | motif file (matrix) |
| 28 \* | A G T C A T C G A C T G C G T A A C G T A G T C C T G A C G T A C G A T A T C G | 1e-7 | -1.781e+01 | 0.28% | 0.08% | 181.7bp (274.7bp) | HNF6(Homeobox)/Liver-Hnf6-ChIP-Seq(ERP000394)/Homer(0.776) More Information \| Similar Motifs Found | motif file (matrix) |
| 29 \* | A C T G A C T G A C T G C G T A C G T A A C G T A C T G A C G T C G T A A C G T A G T C A C T G | 1e-7 | -1.747e+01 | 0.19% | 0.04% | 186.3bp (360.8bp) | TEAD2/MA1121.1/Jaspar(0.720) More Information \| Similar Motifs Found | motif file (matrix) |
| 30 \* | A T C G A G T C A T G C G C T A A C G T C G T A G C T A A C G T C T G A A C G T | 1e-6 | -1.528e+01 | 0.56% | 0.25% | 263.9bp (267.0bp) | ZNF410/MA0752.1/Jaspar(0.671) More Information \| Similar Motifs Found | motif file (matrix) |
| 31 \* | C G T A A G T C A C T G A G T C A G T C C G T A A C G T A G T C C G T A A G T C A C T G A C G T | 1e-6 | -1.489e+01 | 0.12% | 0.02% | 201.1bp (99.7bp) | MLX/MA0663.1/Jaspar(0.659) More Information \| Similar Motifs Found | motif file (matrix) |
| 32 \* | A C T G A G T C A C G T A G T C C G T A C T A G A G T C A C G T A G T C C G T A A C T G A G T C | 1e-5 | -1.377e+01 | 47.60% | 45.15% | 259.4bp (242.4bp) | Zic(Zf)/Cerebellum-ZIC1.2-ChIP-Seq(GSE60731)/Homer(0.562) More Information \| Similar Motifs Found | motif file (matrix) |
